# Ultrasound Sample Entropy Imaging: A New Approach for Evaluating Hepatic Steatosis and Fibrosis

**DOI:** 10.1109/JTEHM.2021.3124937

**Published:** 2021-11-02

**Authors:** Hsien-Jung Chan, Zhuhuang Zhou, Jui Fang, Dar-In Tai, Jeng-Hwei Tseng, Ming-Wei Lai, Bao-Yu Hsieh, Tadashi Yamaguchi, Po-Hsiang Tsui

**Affiliations:** 1 Department of Medical Imaging and Radiological SciencesCollege of Medicine, Chang Gung University 56081 Taoyuan 333323 Taiwan; 2 Department of Biomedical EngineeringFaculty of Environment and LifeBeijing University of Technology 12496 Beijing 100124 China; 3 X-Dimension Center for Medical Research and TranslationChina Medical University Hospital 38020 Taichung 40447 Taiwan; 4 Department of Gastroenterology and HepatologyChang Gung Memorial Hospital at Linkou Taoyuan 333423 Taiwan; 5 Department of Medical Imaging and InterventionChang Gung Memorial Hospital at Linkou Taoyuan 333423 Taiwan; 6 Division of Pediatric GastroenterologyDepartment of PediatricsChang Gung Memorial Hospital at Linkou Taoyuan 333423 Taiwan; 7 Center for Frontier Medical EngineeringChiba University 12737 Chiba 263-8522 Japan; 8 Institute for Radiological Research, Chang Gung University 56081 Taoyuan 333323 Taiwan

**Keywords:** Fatty liver, hepatic steatosis, sample entropy

## Abstract

*Objective:* Hepatic steatosis causes nonalcoholic fatty liver disease and may progress to fibrosis. Ultrasound is the first-line approach to examining hepatic steatosis. Fatty droplets in the liver parenchyma alter ultrasound radiofrequency (RF) signal statistical properties. This study proposes using sample entropy, a measure of irregularity in time-series data determined by the dimension $m$ and tolerance $r$, for ultrasound parametric imaging of hepatic steatosis and fibrosis. *Methods:* Liver donors and patients were enrolled, and their hepatic fat fraction (HFF) ($n =72$), steatosis grade ($n =286$), and fibrosis score ($n =65$) were measured to verify the results of sample entropy imaging using sliding-window processing of ultrasound RF data. *Results:* The sample entropy calculated using $m =$ 4 and $r =0.1$ was highly correlated with the HFF when a small window with a side length of one pulse was used. The areas under the receiver operating characteristic curve for detecting hepatic steatosis that was $\ge $mild, $\ge $moderate, and $\ge $severe were 0.86, 0.90, and 0.88, respectively, and the area was 0.87 for detecting liver fibrosis in individuals with significant steatosis. *Discussion/Conclusions:* Ultrasound sample entropy imaging enables the identification of time-series patterns in RF signals received from the liver. The algorithmic scheme proposed in this study is compatible with general ultrasound pulse-echo systems, allowing clinical fibrosis risk evaluations of individuals with developing hepatic steatosis.

## I. Introduction

Hepatic steatosis, defined as the accumulation of triacylglycerol-rich lipid droplets within hepatocytes (specifically, at least 5% of hepatocytes contain lipid vacuoles), may progress to steatohepatitis, fibrosis, cirrhosis, or hepatocellular carcinoma [1]. Liver biopsy is the gold standard approach for hepatic steatosis diagnosis [2]. Because of the invasiveness, discomfort, and complications of liver biopsy, noninvasive imaging techniques such as computed tomography (CT), magnetic resonance (MR) imaging, MR spectroscopy, and ultrasound, are preferred as alternative assessment tools for hepatic steatosis [3]. However, ionizing radiation limits the practical utility of CT, and high cost reduces the clinical availability of MR techniques. Ultrasound imaging is a cost-effective, real-time, portable technique without ionizing radiation that could serve as a first-line approach to evaluating hepatic steatosis.

Notably, sonography-based diagnosis has substantial drawbacks, including a dependency on backend processing (e.g., image enhancement techniques), reliance on scanning skills, and the possibility of subjective judgments by the examiner. As a result of interobserver and intraobserver variability, the diagnostic sensitivity and specificity of B-scan ultrasonography range from 60% to 94% and from 66% to 95%, respectively [4]. Researchers have focused on developing quantitative ultrasound approaches for characterizing and grading hepatic steatosis to address this variability [5]. The liver parenchyma could be modeled as a scattering medium and therefore form a speckle pattern in an ultrasound B-scan [6]. Because of the randomness of ultrasound backscattering, using statistical distribution models to perform passive parametrization of speckle patterns is a well-accepted method of characterizing the microstructures of tissues (i.e., scatterer arrangements and concentrations) [7], [8]. Currently, the Nakagami and homodyned K (HK) distributions are the two primary general statistical models of ultrasound backscattering [9]. The progression of hepatic steatosis can be quantitatively described using these models with parameters estimated using raw envelope signals (before logarithmic compression) [10]–[12].

One prerequisite of using the distribution models to fit the backscattered statistics is that the statistical properties of the envelope signals used in the analysis must meet the requirements of the model [13], [14]. In practice, this requirement is not necessarily satisfiable because the statistical properties of the envelope waveform are affected by system characteristics and signal postprocessing. Therefore, Shannon entropy, from information theory, was proposed as a method to describe the uncertainty of ultrasound backscattered signals and characterize microstructures of tissues in a more flexible manner [15]. Ultrasound parametric imaging based on Shannon entropy was demonstrated to improve the accuracy of hepatic steatosis assessment compared with that of imaging based on conventional statistical distributions [16] and improved evaluations of metabolic syndrome risks among individuals with hepatic steatosis [17]. The diagnostic performance of Shannon entropy imaging in grading hepatic steatosis is also competitive compared with that of deep learning [18].

Nevertheless, Shannon entropy is calculated using the probability density function (PDF) of the data, which is determined only according to the total information content, without consideration of the time-series causality of ultrasound backscattered signals. In addition, different scattering microstructures may produce the same probability distributions [13], resulting in ambiguity of the physical meaning. Compared with Shannon and other PDF-based entropies, approximate entropy and sample entropy are computational solutions for entropic measures of finite time series; sample entropy was proposed to improve entropy approximation because it is data-length independent and offers better computational consistency during the determination of information regularity based on patterns in the time-series data [19]. This implies that the use of sample entropy can achieve more accurate hepatic steatosis characterization compared with that achieved by methods that involve analysis of the PDF of the envelope according to pattern similarities in the time series of raw radiofrequency (RF) signals obtained after beamforming. Moreover, this also implies that the use of sample entropy can provide more information regarding the physical interactions between the incident wave and acoustic scatterers.

This study explored the utility of sample entropy in ultrasound parametric imaging and grading of hepatic steatosis and fibrosis. In the following sections, we introduce the theoretical background of sample entropy and explain how we designed an algorithmic scheme and acquired clinical data for validation of the proposed method. The results revealed that the diagnostic performance of sample entropy calculated using raw RF signals was superior to that of conventional PDF-based Shannon entropy in grading ultrasound images of hepatic steatosis. Ultrasound sample entropy imaging could also detect liver fibrosis in individuals with significant hepatic steatosis.

## II. Materials and Methods

### A. Theoretical Background

Sample entropy is a measure of the irregularity of a unidimensional series and is defined as the negative natural logarithm of the conditional probability that a subseries remains similar when its data length increases, where self-matches are not included in calculating the probability. Details of the algorithm can be found in a previous study [19] and are briefly described as follows.

We assume time-series data of length $N$ with a constant time interval $\tau $ for an ultrasound backscattered RF signal, as given by

\begin{equation*} X=\left \{{x_{1}, x_{2}, x_{3}, \ldots, x_{N}}\right \}.\tag{1}\end{equation*}

The conventional Shannon entropy (denoted by ShanEn) is calculated as [16]

\begin{equation*} {~\text {ShanEn }}=-\sum \nolimits _{i=1}^{n} w\left ({x_{i}}\right) log _{2}\left [{w\left ({x_{i}}\right)}\right],\tag{2}\end{equation*}

where $x_{\mathrm {i}}$ are the discrete random variables representing the backscattered signals, $w(x_{\mathrm {i}})$ represents the probability value of a signal in bin $i$, and $n$ indicates the number of bins. Template vectors (i.e., subseries) with length $m$ (the dimension parameter) are

\begin{equation*} X_{m}(i)=\left \{{x_{i}, x_{i+1}, x_{i+2}, \ldots, x_{i+m-1}}\right \},\tag{3}\end{equation*}

and their distance function (based on the Chebyshev distance) is

\begin{equation*} d\left [{X_{m}(i), X_{m}(j)}\right],\quad i \neq j.\tag{4}\end{equation*}

The sample entropy (denoted by SampEn) is then defined as

**FIGURE 1.**
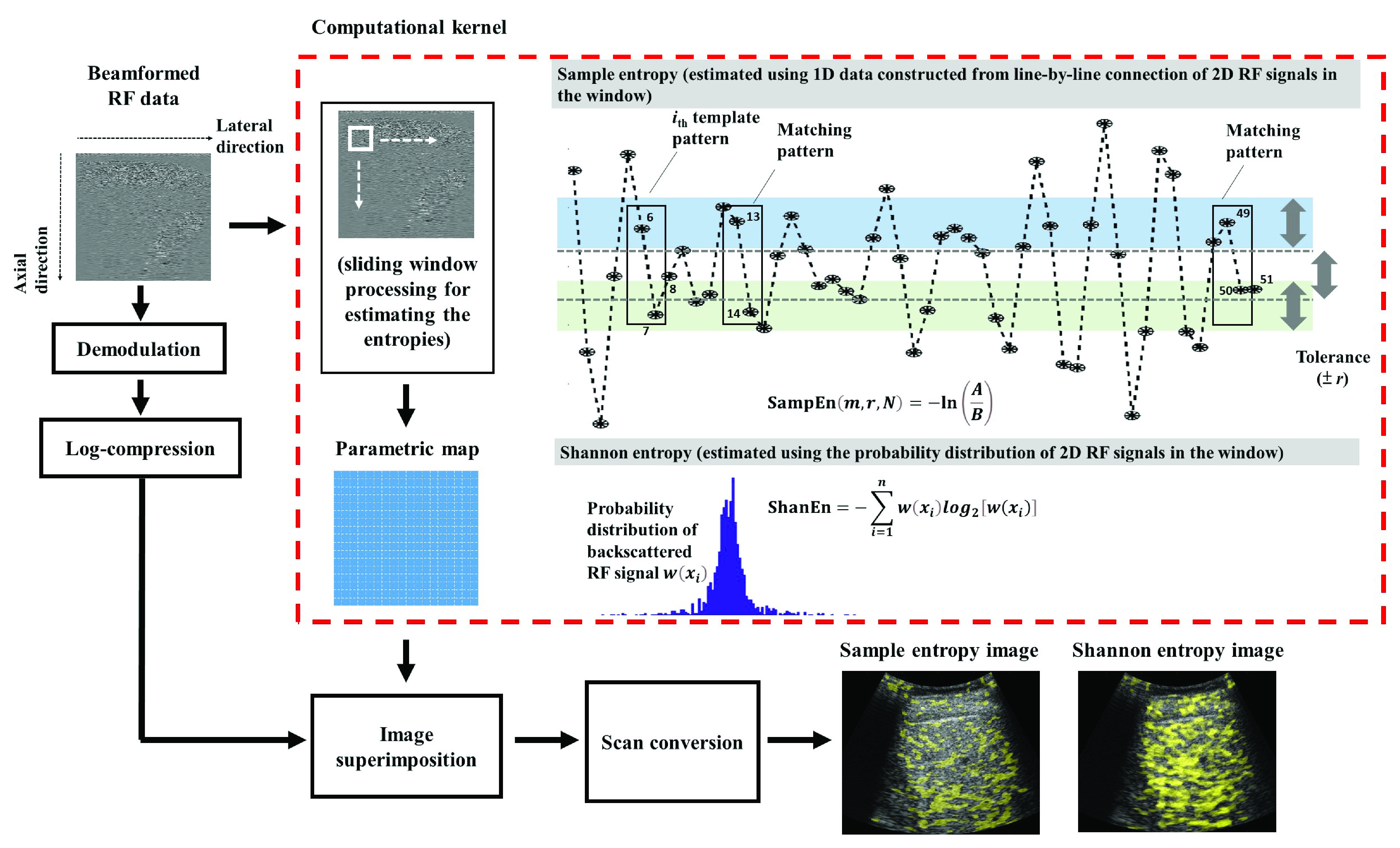
Algorithmic scheme for ultrasound parametric imaging using sample and Shannon entropies. This illustration presents an example of the sample entropy calculation process with the dimension $m =2$. The data points $x_{6}$ and $x_{7}$ are used as the $i_{\mathrm {th}}$ template pattern ($i =6$). The 2-point sequences $x_{13}$, $x_{14}$ and $x_{49}$, $x_{50}$ are matched, and $x_{49}$, $x_{50}$, $x_{51}$ is a 3-point matched sequence. In this case, SampEn = −ln (1/2).

\begin{equation*} \mathrm {SampEn}(m, r, N)=-\ln \left ({\frac {A}{B}}\right),\tag{5}\end{equation*}

where $r$ is the tolerance value, $A$ represents the number of template vector pairs for which $d\left [{X_{m+1}(i), X_{m+1}(j)}\right] < r$, and $B$ is the number of template vector pairs for which $d\left [{X_{m}(i), X_{m}(j)}\right] < r$. The tolerance value $r$ is a percentage multiplied by the standard deviation of the original sequence; thus, the time series should be normalized before analysis [20]. Sample entropy approaches zero for signals with highly periodic patterns and increases for irregular signals [20], [21]. An example of the sample entropy calculation process is provided in the top-right corner of Fig. 1.

### B. Study Populations

This study received approval from the Institutional Review Board (IRB) of Chang Gung Memorial Hospital for the reuse of previously collected data [16] and the enrollment of new participants for research purposes (IRB no. 201601928B0; approval date: Feb. 7, 2017). All participants signed informed consent forms, and experimental methods conformed with the approved guidelines. Liver donors were enrolled in Group I ($n =72$). Patients scheduled to undergo liver biopsy due to confirmed chronic hepatitis B infection or partial liver resection were enrolled in Groups II ($n =286$) and III ($n =65$). The details are described below.

Anthropometric and biochemical examinations (after overnight fasting for 8 h) were performed to measure the body mass index, aspartate aminotransferase level, and alanine aminotransferase level of each patient in each group. The hepatic fat fraction (HFF) of the liver (right lobe) in each liver donor in Group I was measured through hydrogen-1 ($^{1}\text{H}$) proton MR spectroscopy (i.e., $^{1}\text{H}$-MRS) (GE Signa HDXT, Waukesha, WI, USA), a well-recognized noninvasive approach that accurately quantifies hepatic steatosis [22]. The HFF was defined as (fat mass)/(fat mass + water mass) and expressed as a percentage. For patients in Group II, we obtained specimens from the right liver lobe by adopting an intercostal approach and sent them to the department of pathology for histological examination. Hepatic steatosis was graded according to the percentage of involved hepatocytes [23]: normal (< 5%), mild (5%–33%), moderate (33%–66%), and severe (>66%). Considering that hepatic steatosis is a risk factor for nonalcoholic steatohepatitis, which causes liver fibrosis [23], additional 65 patients histologically graded as having moderate or severe hepatic steatosis were included in Group III to undergo METAVIR scoring; these scores were classified as F0, no fibrosis; F1, portal fibrosis with no septa; F2, portal fibrosis with few septa; F3, bridging fibrosis with many septa; and F4, cirrhosis (nodular stage) [23]. The patients’ demographic data are summarized in Tables 1 and 2. We performed $^{1}\text{H}$-MRS and liver biopsy examinations after abdominal ultrasound scanning.

**TABLE 1:** Demographic Data of Patients Enrolled in Groups I and II

Characteristics	Group I	Group II
Male/Female	27/45	160/126
Age, years		
Mean ± standard deviation (range)	33 ± 9.4 (18 – 55)	51 ± 12.5 (19 – 79)
Median	31	52
BMI, kg/m^2^		
Mean ± standard deviation (range)	23.3 ± 3.2 (17.3 – 30.4)	25.9 ± 4.0 (16.8 – 41.2)
Median	22.9	25.7
AST, U/L		
Mean ± standard deviation (range)	24.3 ± 10.1 (15 – 66)	73.5 ± 80.8 (3.5 – 901)
Median	21	51
ALT, U/L		
Mean ± standard deviation (range)	22.3 ± 19.5 (5 – 114)	110.6 ± 155.9 (8 – 2034)
Median	15	77
Hepatic steatosis grade, no. of patients		
Normal	–	97
Mild	–	77
Moderate	–	43
Severe	–	69

Unless otherwise noted, data are expressed as numbers of patients. BMI: body mass index, AST: aspartate aminotransferase, ALT: alanine aminotransferase. Normal AST levels for female and male patients are less than 35 and 50 U/L, respectively. Normal ALT levels for female and male patients are less than 19 and 30 U/L, respectively.

**TABLE 2:** Demographic Data of Patients in Group III (Individuals With Moderate or Severe Hepatic Steatosis Who Had an METAVIR Score)

Characteristics	Group III
Male/Female	44/21
Age, years	
Mean ± standard deviation (range)	53.26 ± 10.76 (35 - 74)
Median	54
BMI, kg/m^2^	
Mean ± standard deviation (range)	27.61 ± 3.40 (21.37 - 37.83)
Median	27.82
AST, U/L	
Mean ± standard deviation (range)	74.37 ± 70.91 (25 - 507)
Median	53
ALT, U/L	
Mean ± standard deviation (range)	97.07 ± 91.82 (20 - 595)
Median	74
Histological fibrosis grade, no. of patients	
F0	7
F1	14
F2	9
F3	16
F4	19

Unless otherwise noted, data are expressed as numbers of patients. BMI: body mass index, AST: aspartate aminotransferase, ALT: alanine aminotransferase. Normal AST levels for female and male patients are less than 35 and 50 U/L, respectively. Normal ALT levels for female and male patients are less than 19 and 30 U/L, respectively.

### C. Ultrasound Data Acquisition and Analysis

The subjects underwent standard-care abdominal ultrasound. We obtained three liver parenchyma scans for each patient by using the intercostal scanning approach [24], and major vessels, structures, and acoustic shadowing artifacts were excluded during scanning [25]. A clinical ultrasound scanner (Model 3000, Terason, Burlington, MA, USA) equipped with a convex array transducer with a 3-MHz central frequency (Model 5C2A, Terason) was used. The gain index in the software of the Terason scanner is adjustable and was set at 6, which corresponds to the gain of 33 dB according to the calibrations performed in the previous study [26]. The system-default time-gain compensation curve in terms of an exponentially increasing function constructed using an attenuation coefficient of 0.3 dB/MHz-cm (a conservative assumption generally used in commercial ultrasound systems) was used to reduce the acoustic attenuation effect. The pulse length (PL) obtained from the pulse-echo measurement of the transducer was approximately 2.3 mm, and the focus and depth for imaging were set at 4 and 8 cm, respectively. For each scan, the imaging data were saved in ULT file format. No apodization, filtering, and any kind of signal processing were used during ultrasound transmission and data acquisition. The software kit provided by Terason was then used to convert the image data into raw beamformed RF signals (sampling rate: 12 MHz; 128 scan lines) for offline analysis (Fig. 1).

The absolute value of the Hilbert transform of each RF signal was taken to form the envelope image, which was then compressed through logarithmic computation to display the brightness mode (B-mode) image at a dynamic range (DR) of 40 dB. Sliding-window processing, a common processing technique for generating an ultrasound parametric image [15]–[17], was applied to the RF data (not the envelope image) for entropy parametric imaging. According to the line-by-line connection basis, two-dimensional (2D) RF signals within a square window were converted into one-dimensional (1D) time-series data for estimating local entropy values, which were stored as pixels corresponding to the window location. The window was moved across the range of RF data in an overlapping fashion at fixed distances of 50% of the window side length (WSL) to balance image quality and computational efficiency [27]. Different WSLs (WSL: 1–3 PL), dimension parameters ($m$: 1–10), and tolerance values ($r$: 0.05–0.5) were used for sample entropy imaging. Conventional Shannon entropy imaging (WSL = 1 PL; bins = 200) [15], [16] was also performed for comparison. Finally, both the Shannon and sample entropy images underwent two-dimensional interpolation to compensate for loss in image size caused by the sliding-window processing method [28], and the entropy images were merged with the corresponding B-mode images for display purposes (DRs of Shannon and sample images were set at 3.8–4.2 and 1.7–3.0, respectively). The regions of interest (ROI) on the B-scan of the liver parenchyma were manually outlined by an experienced radiologist, and the pixel values in both Shannon and sample entropy images corresponding to the ROI were used to calculate the average entropy values for quantitative analysis.

### D. Statistical Analysis

For Group I, the Pearson correlation coefficients $r_{\mathrm {p}}$ between the sample entropy and the HFF obtained from using different WSLs, $m$, and $r$ were calculated to determine the optimal combination of computational setting to maximize $r_{\mathrm {p}}$. For Groups II and III, the entropy values as a function of steatosis grade and METAVIR score were expressed using box plots overlaid with dot plots. The Spearman rank correlation $r_{\mathrm {s}}$ was also calculated. To evaluate the diagnostic performance, receiver operating characteristic (ROC) curve analysis was performed. The area under the ROC curve (AUROC), sensitivity, specificity, accuracy, and other statistical results were reported. The DeLong test was used to compare ROC curves and identify significant differences in diagnostic performance. All statistical analyses were performed using SigmaPlot (version 12.0, Systat Software, Inc., CA, USA). Statistical significance was set at $p < 0.05$.

## III. Results

**FIGURE 2.**
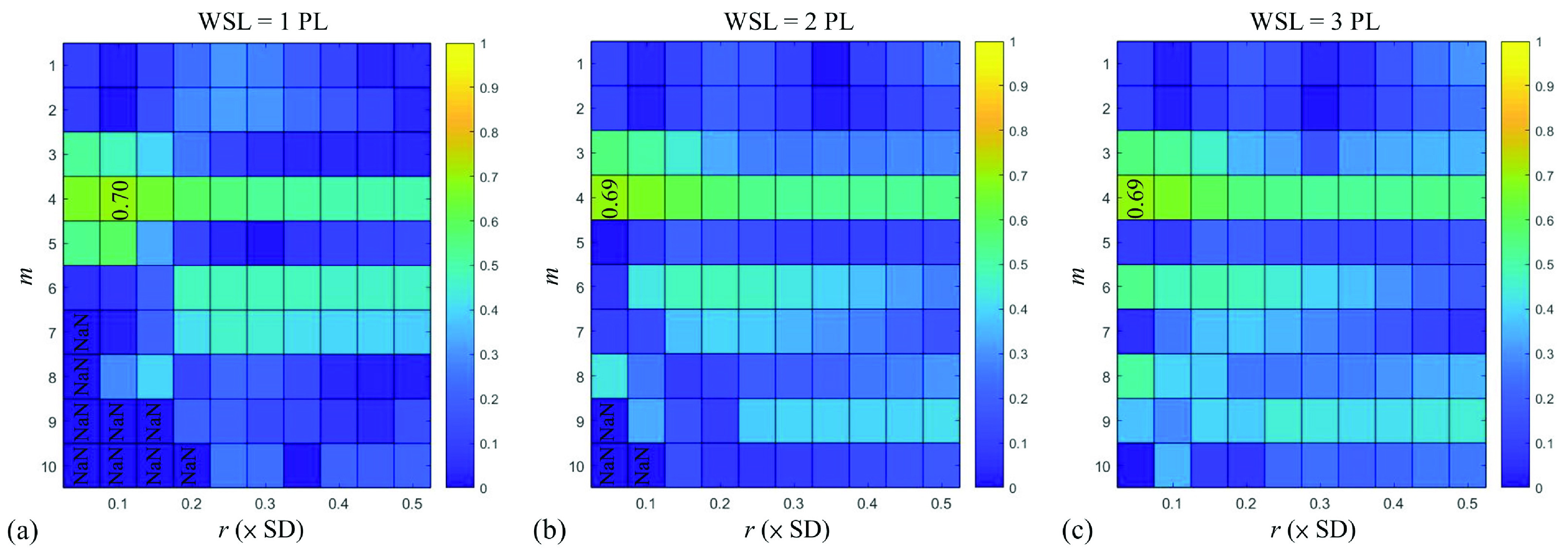
Correlation matrixes obtained under different computational settings to demonstrate the association between sample entropy and the HFF values for Group I, suggesting that WSL = 1 PL, $m =4$, and $r =0.1$ are optimal computational values for the calculation of sample entropy.

**FIGURE 3.**
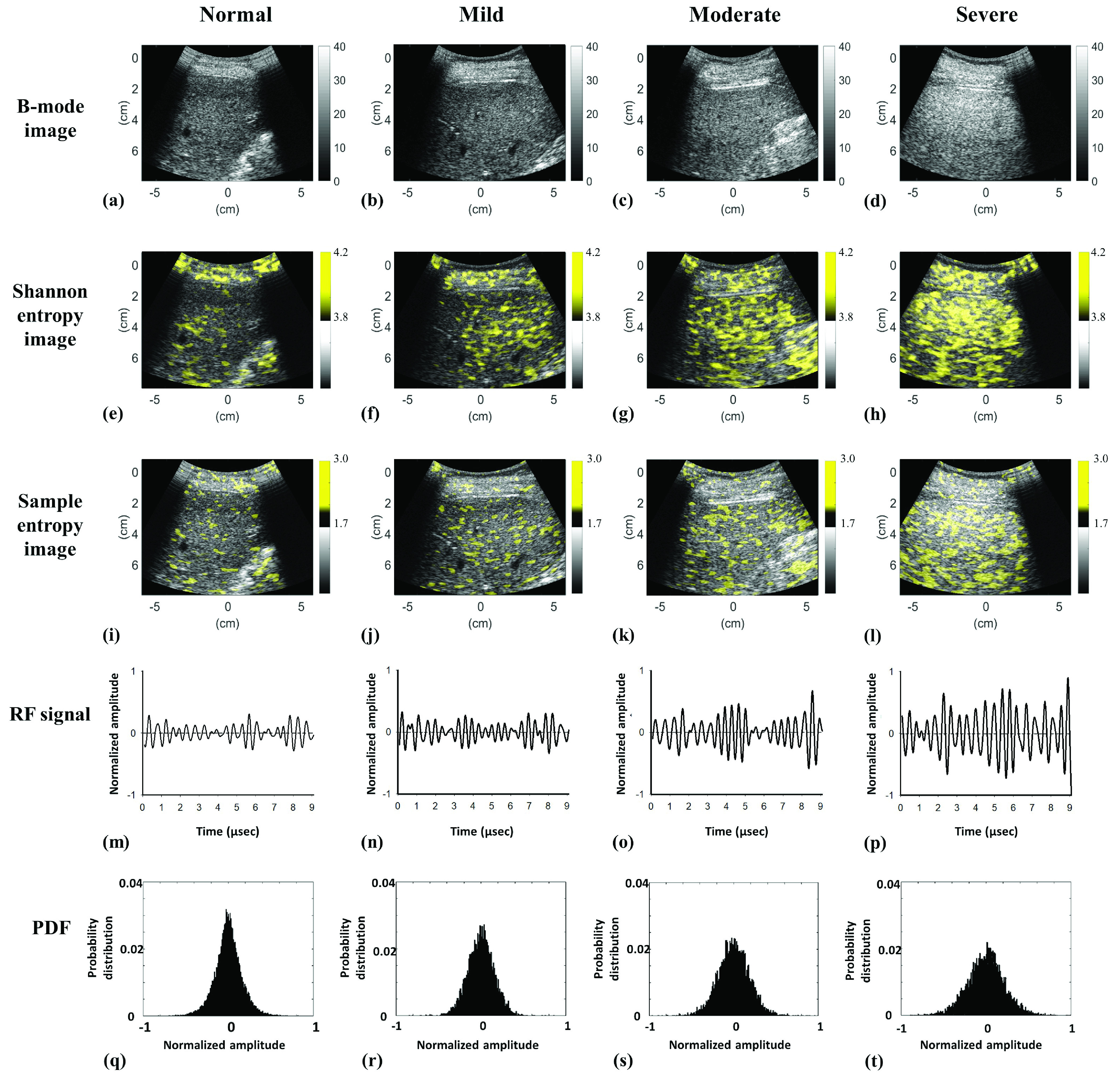
Typical B-scan, entropy images, RF signals, and PDFs at various HFFs. (a)–(d) B-mode images at the HFF values of 0.78%, 11.27%, 28.72%, and 44.66%, respectively; (e)–(h) Shannon entropy images corresponding to (a)–(d); (i)–(l) sample entropy images corresponding to (a)–(d); (m)–(p) representative RF signals corresponding to (a)–(d); (q)–(t) PDFs corresponding to (m)–(p).

**FIGURE 4.**
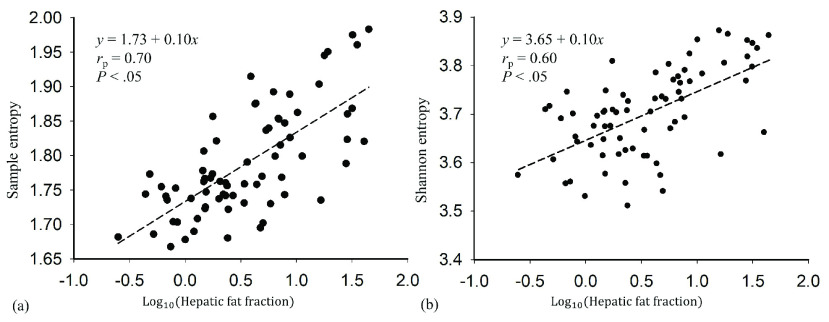
Relationships between entropy values and HFFs. Both types of entropy were proportional to the logarithmic transform of the HFF; sample entropy was more strongly correlated with the HFF ($r_{\mathrm {p}} =0.70$; $p < 0.05$).

Fig. 2 presents the correlation matrixes of the data in Group I to visualize the $r_{\mathrm {p}}$ values for correlations between the HFF and the sample entropy values obtained using different combinations of computational settings. For each WSL, the correlation matrixes exhibited similar patterns and distributions. The maximum $r_{\mathrm {p}}$ between the HFF and the sample entropy was observed at $m =4$ and $r$ from 0.05 to 0.1. Using a small window improves the spatial resolution of a parametric image; therefore, the values WSL = 1 PL, $m =4$, and $r =0.1$ were used for sample entropy imaging in the following analysis. Fig. 3(a)–(l) shows typical B-mode, Shannon entropy, and sample entropy images for various HFF values. The brightness and distribution of the false coloring of Shannon entropy images varied at higher HFFs; the same phenomenon was observed in sample entropy images. Fig. 3(m)–(t) presents typical RF signals and the corresponding PDFs used for sample and Shannon entropy imaging, respectively. The amplitude of RF data strengthened with increasing the HFF, leading to an increasing in the PDF width. Fig. 4 further illustrates the entropy values as a function of HFF. The $r_{\mathrm {p}}$ value of the sample entropy was proportional to the logarithmic transform of the HFF ($r_{\mathrm {p}} =0.70$; $p < 0.05$), as was the $r_{\mathrm {p}}$ value of the Shannon entropy ($r_{\mathrm {p}} =0.60$; $p < 0.05$).

**FIGURE 5.**
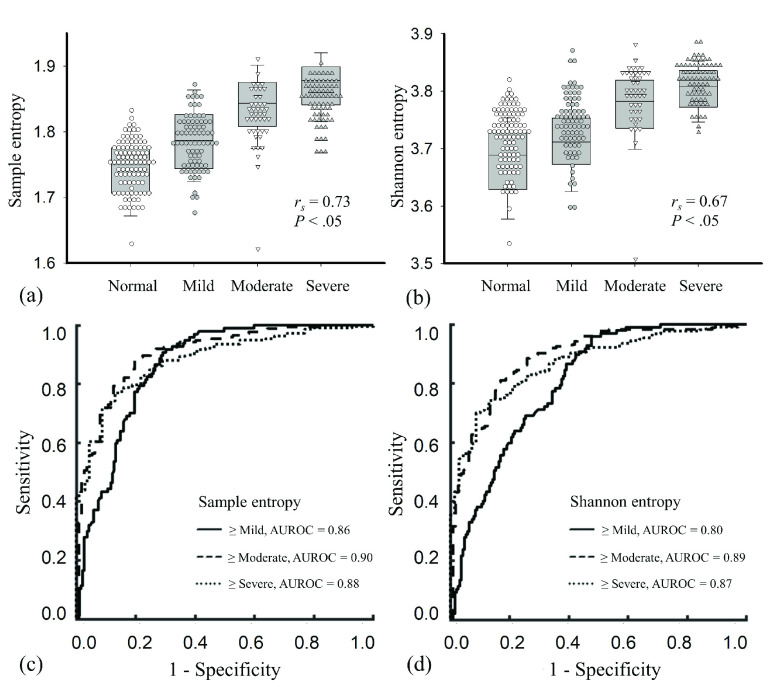
(a) and (b) Entropy values as a function of hepatic steatosis grade; (c) and (d) ROC curves for using sample and Shannon entropies for hepatic steatosis diagnosis. Sample entropy outperformed Shannon entropy in ultrasound parametric imaging for the grading of early hepatic steatosis.

Fig. 5(a) and (b) display the box and dot plots obtained from the Group II data for sample entropy and Shannon entropy values stratified according to the grade of hepatic steatosis. From normal to severe grades of hepatic steatosis, the median and interquartile range (IQR) of the sample entropy increased from 1.75 (IQR: 0.07) to 1.87 (IQR: 0.06) ($r_{\mathrm {s}} =0.73$; $p < 0.05$), and those of the Shannon entropy increased from 3.69 (IQR: 0.10) to 3.81 (IQR: 0.06) ($r_{\mathrm {s}} =0.67$; $p < 0.05$). Fig. 5(c) and (d) display the ROC curves obtained from using sample entropies and Shannon entropies for grading hepatic steatosis. The AUROCs of (SampEn, ShanEN) for classifying hepatic steatosis as $\ge $mild, $\ge $moderate, and $\ge $severe were (0.86, 0.80) ($p < 0.05$), (0.90, 0.89) ($p >0.05$), and (0.88, 0.87) ($p >0.05$), respectively. Detailed statistical data of performance evaluations are summarized in Table 3.

**TABLE 3:** Performance Metrics for Using Shannon and Sample Entropies to Grade Hepatic Steatosis as $\ge$ Mild, $\ge$ Moderate, and $\ge$ Severe

	$\ge $Mild		$\ge $Moderate		$\ge $Severe	
Parameter	Shannon entropy	Sample entropy	Shannon entropy	Sample entropy	Shannon entropy	Sample entropy
Cutoff value	3.71	1.78	3.74	1.82	3.75	1.82
Sensitivity, %	69.07	79.38	80.50	88.51	74.19	76.96
Specificity, %	74.60	78.84	83.93	80.36	86.96	86.96
Accuracy, %	72.73	79.02	81.82	85.31	77.27	79.37
LR+	2.72	3.75	5.01	4.51	5.69	5.90
LR−	0.41	0.26	0.23	0.14	0.30	0.26
PPV, %	58.26	65.81	88.61	87.50	94.71	94.89
NPV, %	82.46	88.17	73.44	81.82	51.72	54.55
AUROC	0.80	0.86	0.89	0.90	0.87	0.88
ROC comparison by DeLong test						
Area Difference	0.060		0.016		0.014	
SE	0.016		0.013		0.015	
Z Value	13.61		1.61		0.89	
P Value	0.000		0.204		0.346	

LR== positive likelihood ratio; LR− = negative likelihood ratio; PPV = positive predictive value; NPV = negative predictive value; AUROC = area under the receiver operating characteristic curve; SE = standard error.

**FIGURE 6.**
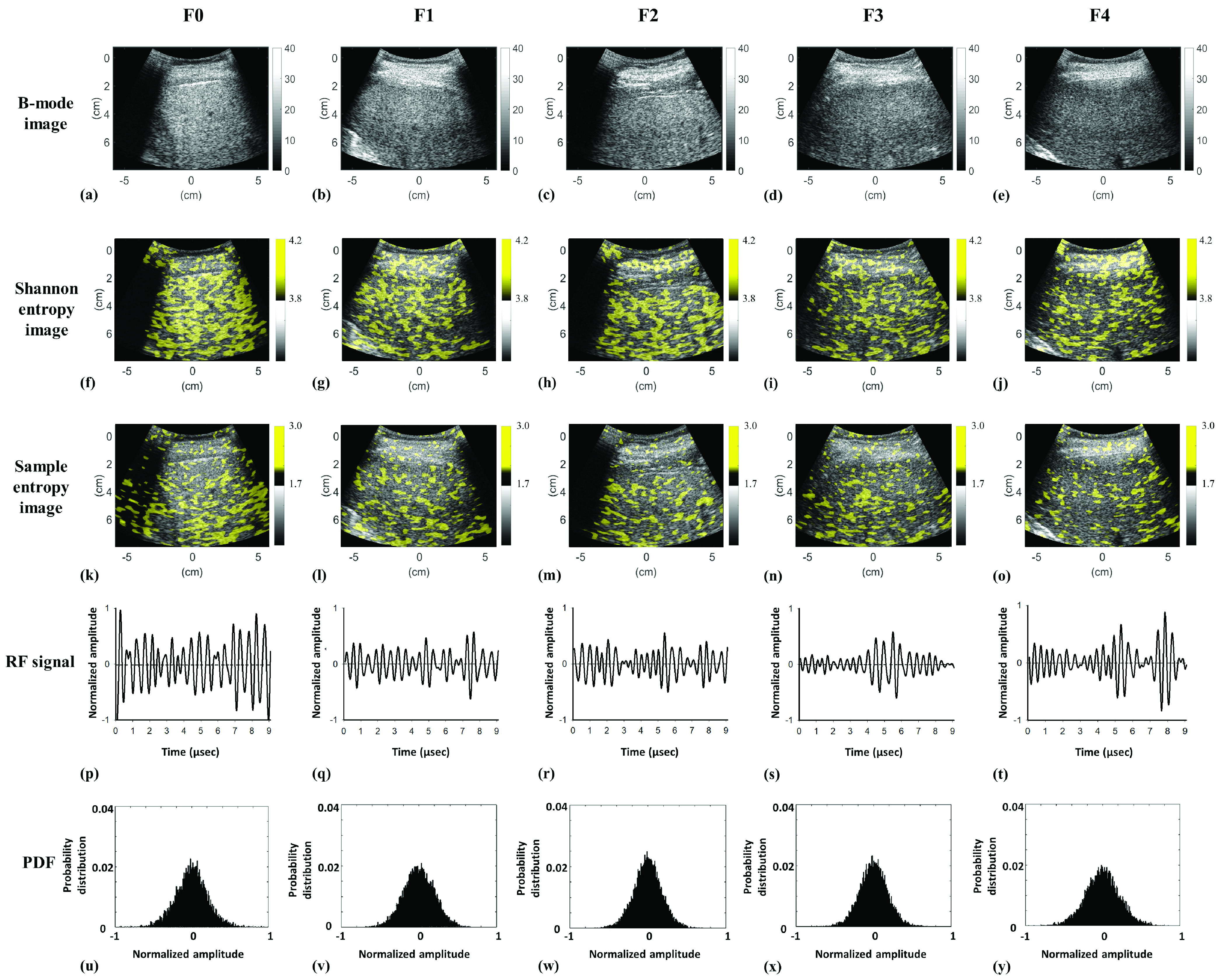
Typical B-scan, entropy images, RF signals, and PDFs for different stages of liver fibrosis in patients with severe hepatic steatosis. (a)–(e) B-mode images at F0–F4, respectively; (f)–(j) Shannon entropy images corresponding to (a)–(e); (k)–(o) sample entropy images corresponding to (a)–(e); (p)–(t) representative RF signals corresponding to (a)–(e); (u)–(y) PDFs corresponding to (p)–(t).

**FIGURE 7.**
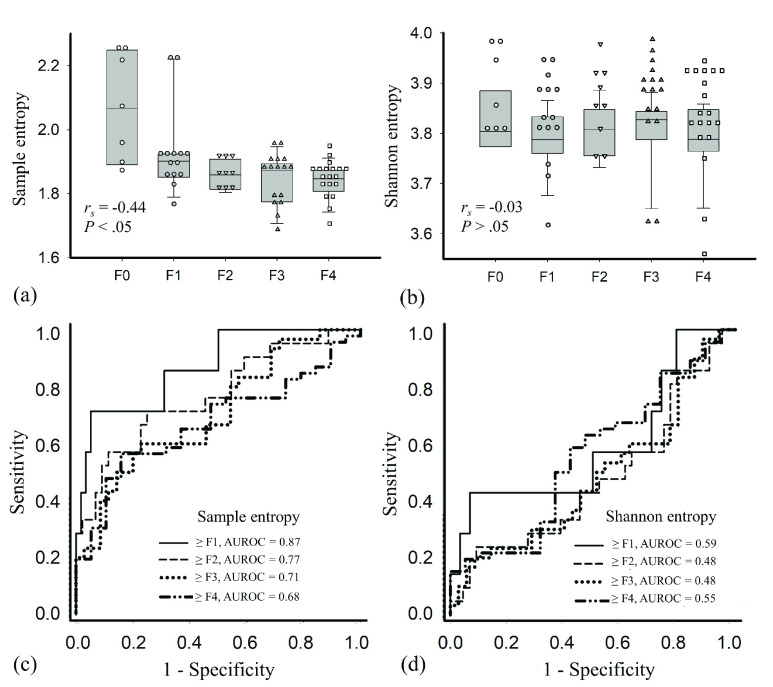
(a) Sample and (b) Shannon entropy values as a function of liver fibrosis score; (c) Sample and (d) Shannon entropy ROC curves for liver fibrosis diagnosis. Sample entropy outperformed Shannon entropy in the detection of liver fibrosis in patients with moderate-to-severe hepatic steatosis.

Fig. 6(a)–(o) presents typical B-mode, sample entropy, and Shannon entropy images at different stages of liver fibrosis for patients with severe hepatic steatosis (Group III). When sample entropy imaging was used, the brightness of ultrasound entropy images was lower for patients with greater liver fibrosis severity; however, conventional Shannon entropy imaging did not reveal a difference in brightness between controls and individuals with liver fibrosis. Fig. 6(p)–(y) presents typical RF signals and the corresponding PDFs at different fibrosis stages. The echo amplitude of RF data for the steatosis case without liver fibrosis was relatively strong, and that for fibrosis cases became weak and exhibited a higher degree of variations in signal amplitude. Fig. 7(a) and (b) present box and dot plots for Group III quantitatively confirming these observations. The median sample entropy decreased from 2.07 (IQR: 0.31) to 1.85 (IQR: 0.06) as the METAVIR score increased from F0 to F4 ($r_{\mathrm {s}} = -0.44$; $p < 0.05$). The Shannon entropy values were widely distributed and had no significant association with the METAVIR score ($r_{\mathrm {s}} = -0.03$; $p >0.05$). Fig. 7(c) and (d) display the ROC curves obtained from using sample and Shannon entropies to detect liver fibrosis. The AUROCs of (SampEn, ShanEN) for classifying liver fibrosis as $\ge $F1, $\ge $F2, $\ge $F3, and $\ge $F4 were (0.87, 0.59) ($p >0.05$), (0.77, 0.48) ($p < 0.05$), (0.71, 0.48) ($p < 0.05$), and (0.68, 0.55) ($p >0.05$), respectively. Performance metrics are shown in Table 4. Sample entropy outperformed Shannon entropy for the detection of liver fibrosis in patients with significant hepatic steatosis.

**TABLE 4:** Performance Metrics for Using Shannon and Sample Entropies to Detect Liver Fibrosis in Individuals With Moderate-to-Severe Hepatic Steatosis

	$\ge $F1		$\ge $F2		$\ge $F3		$\ge $F4	
Parameter	Shannon entropy	Sample entropy	Shannon entropy	Sample entropy	Shannon entropy	Sample entropy	Shannon entropy	Sample entropy
Cutoff value	3.86	1.94	3.78	1.88	3.77	1.88	3.79	1.87
Sensitivity, %	93.10	94.83	52.38	75.00	40.00	77.14	57.89	84.21
Specificity, %	42.86	71.43	61.36	71.43	77.14	60.00	58.70	56.52
Accuracy, %	87.69	92.31	58.46	73.85	60.00	69.23	58.46	64.62
LR+	1.63	3.32	1.36	2.63	1.75	1.93	1.40	1.94
LR−	0.16	0.07	0.78	0.35	0.78	0.38	0.72	0.28
PPV, %	93.10	96.49	39.29	84.62	60.00	69.23	36.67	44.44
NPV, %	42.86	62.50	72.97	57.69	60.00	69.23	77.14	89.66
AUROC	0.59	0.87	0.48	0.77	0.48	0.71	0.55	0.68
ROC comparison by DeLong test								
Area Difference	0.276		0.292		0.224		0.120	
SE	0.196		0.082		0.072		0.072	
Z Value	1.97		12.67		9.58		2.78	
P Value	0.160		0.000		0.002		0.095	

LR+ = positive likelihood ratio; LR − + negative likelihood ratio; PPV = positive predictive value; NPV = negative predictive value; AUROC = area under the receiver operating characteristic curve; SE = standard error.

## IV. Discussion

### A. Significance of This Study

This study explored the feasibility of using sample entropy in ultrasound parametric imaging of hepatic steatosis and fibrosis. We compared the diagnostic performance of sample entropy with that of conventional Shannon entropy by conducting clinical experiments. Raw RF signals obtained after beamforming were used for the entropy analysis. The results in Group I suggested that a value set of (WSL = 1 PL, $m =4$, $r =0.1$) maximizes the strength of the correlation between sample entropy and the HFF. With this computational setting, sample entropy has two advantages over Shannon entropy in clinical evaluations of hepatic steatosis and liver fibrosis. First, sample entropy outperformed Shannon entropy for the grading of early hepatic steatosis ($\ge $mild) through ultrasound parametric imaging, as supported by the Group II results (Table 3). Second, compared with Shannon entropy that failed in liver fibrosis detection, ultrasound sample entropy imaging allowed identification of coexisting liver fibrosis ($\ge $F1) in patients with moderate-to-severe hepatic steatosis, as demonstrated by the findings for Group III (Table 4). This study confirms that sample entropy provides improved diagnostic performance in detecting early hepatic steatosis and identifying coexisting liver fibrosis as steatosis progresses to significant stages.

### B. Effects of Hepatic Steatosis and Fibrosis on Entropy

Entropy values increased with the progression of hepatic steatosis, representing an increase in the irregularity of ultrasound backscattered RF signals, which could be specifically comprehended and interpreted by changes in the signal waveform and PDF [15]–[17], [29]. In brief, the primary acoustic scatterers in normal liver parenchyma include hepatocytes and lobules, which are diffuse and coherent components, respectively [6]. In a fat-infiltrated liver, a single large fat droplet in a hepatocyte can push the nucleus to the periphery (i.e., macrovesicular steatosis) [30]. The single large fat droplet in macrovesicular steatosis is believed to form from the fusion of multiple small- to medium-sized fat droplets [31]. These fat-infiltrated hepatocytes are newly added acoustic scatterers with not only increased density but also increased scattering cross-section areas to strengthen constructive wave interference, increasing ultrasound signal amplitudes, broadening PDFs, and elevating entropies (Fig. 3). However, the effect of liver fibrosis on the time-series patterns of ultrasound signals differs from that of hepatic steatosis. A fat-infiltrated liver with coexisting liver fibrosis could be treated as an inhomogeneous tissue with various scattering cross-sections of the scatterers [32]. Thus, such an inhomogeneity of tissue tends to result in destructive wave interference, significant variations in amplitude, and narrow PDFs for the time-domain signals [33] (Fig. 6), reducing the informational complexity corresponding to a decrease in entropy.

Notably, conventional Shannon entropy was not associated with liver fibrosis severity in the patients with hepatic steatosis; however, the sample entropy was sensitive to liver fibrosis detection. In the next part, a detailed comparison of sample and Shannon entropies reveals some reasons for this difference.

### C. Comparison Between Sample and Shannon Entropies

Initially, an algorithmic scheme based on Fourier analysis was proposed to establish the PDF of ultrasound RF data to calculate Shannon entropy [34]–[36]. The statistical histogram was then used as a less computationally complex alternative method to reconstruct the PDF of ultrasound signals, increasing the practical applicability of Shannon entropy [16]–[18], [29], [37]. A common problem for PDF-based entropies is that the probability distributions of ultrasound RF signals may be identical for different scattering microstructures, causing ambiguity in the physical meaning of ultrasound backscattering. For example, a tissue with few scatterers or a homogeneous medium with strong scatterers causes variation in the size of the scattering cross-section, resulting in the same type of backscattered statistics [13]. In addition, because fibrous structures in the liver are acoustically weaker scatterers than are fatty droplets, the formation of backscattered signals tends to be dominated by fatty droplets [32]. For these reasons, changes in the PDF of the backscattered signals caused by liver fibrosis may not be apparent, causing Shannon entropy and other PDF-based entropies to exhibit information-mixing problems that inhibit the detection of liver fibrosis. Comparatively, sample entropy is an adaptive analysis method for measuring the irregularity of raw time-series data without information mixing or the information loss caused by the transformation of the signal into a probability distribution. Also, sample entropy is constructed directly using RF signals, which are generated from the interaction of ultrasound with the liver tissue; thus, the physical interpretations of sample entropy imaging may be more relevant to changes in the microstructures. As supported by Fig. 6, even if in a challenging condition that steatosis and fibrosis coexisted in the liver, the time-domain RF signal appeared to be more sensitive to variations in the scatterer properties, which were not reflected by the PDF. These advantages likely explain why sample entropy outperformed Shannon entropy in grading early hepatic steatosis for the detection of liver fibrosis in patients with significant hepatic steatosis.

The $p$ value obtained from comparing the sample and Shannon entropy ROC curves for the detection of liver fibrosis $\ge $ F1 was larger than 0.05 despite the AUROC of the sample entropy being larger than that of the Shannon entropy. The lack of statistical significance is probably attributable to the inclusion of only seven controls in Group III; a limited sample size influences statistical comparisons using the DeLong test.

### D. Effects of Computational Settings on Sample Entropy

The ultrasound parametric imaging of sample entropy has three computational settings: WSL, $m$, and $r$. As shown in Fig. 2, the WSL had a relatively small effect on the sample entropy estimation; one PL was sufficient to observe the characteristics of oscillation and irregularity in ultrasound RF signals. However, the sample entropy was sensitive to the dimension $m$ and tolerance $r$. The tolerance value $r$ may be determined empirically for different practical applications and purposes and for different types of received signals because it measures the regularity, or frequency, of occurrence of patterns similar to a given template of a given length (i.e., the dimension $m$). Therefore, a universal tolerance value does not exist and must be calibrated for different diagnostic requirements. In contrast to $r$, the threshold magnitude for signal distances, the dimension parameter $m$ is used to determine the length of a given time series for pattern similarity comparisons. The ultrasound backscattered RF signals used in the analysis are discrete time-series data obtained from sampling continuous signals through analog-to-digital conversion. Thus, an effective dimension value $m$ should be close to the number of sampling points in one cycle to ensure that the similarity comparison is based on one wave pattern. In this study, the central frequency of the transducer was 3 MHz, and the sample rate of the system was 12 MHz; therefore, one cycle consisted of approximately four sampling points. This cycle length may explain why the strongest correlation between sample entropy and HFF was observed at $m =4$.

### E. Clinical and Translational Impacts of This Study

Currently, the FibroScan device (Echosens, Paris, France) based on vibration-controlled transient elastography has been recognized as a noninvasive technique in the diagnosis of hepatic steatosis and liver fibrosis [38]. FibroScan measures the shear wave velocity, which is then converted into the stiffness expressed in kilopascals for liver fibrosis detection; it also measures ultrasound attenuation to calculate controlled attenuation parameter (CAP) for hepatic steatosis assessment. However, FibroScan only assesses part of the liver without imaging guidance [38]. Some factors affect the reliability of stiffness measurements by FibroScan, such as inflammation, obesity, food intake, and operator training [39], [40]. Body mass index and the thickness of subcutaneous adipose tissue also influence CAP [41], which has worse performance in detecting severe steatosis [40]. Importantly, CAP (i.e., attenuation) physically reflects the redistribution of the acoustic energy caused by the effects of wave absorption in the liver [24]. Such a physical meaning for CAP is associated with the viscoelastic properties of the liver [24] and does not exactly correspond to a pathological change in the microstructures of the liver parenchyma (i.e., the criteria used for diagnosing steatosis) [40].

In comparison, ultrasound backscattered statistics analysis has some advantages to complement FibroScan in the management of liver disease. First, ultrasound backscattering varies with changes in microstructures, and therefore its association with pathological changes is more significant accordingly [16], [25]. Second, ultrasound backscattering is less affected by the inflammation effect [25], implying that backscattered statistics may be applicable to examinations on patients with chronic inflammation in the liver (e.g., steatohepatitis). Third, the algorithmic scheme of ultrasound backscattered statistics parametric imaging is totally compatible with ultrasound pulse-echo imaging systems, providing opportunities to upgrade general B-scan machines as the alternative of FibroScan to produce a value-added and image-guided diagnosis. Notably, as mentioned in Introduction, ultrasound parametric imaging using the Nakagami and HK distributions has been applied to clinical assessment of hepatic steatosis. It has been reported that the AUROCs for using Nakagami and HK imaging to detect hepatic steatosis ($\ge $mild, $\ge $moderate, $\ge $severe) were (0.75, 0.82, 0.82) and (0.76, 0.80, 0.83), respectively [11], [12], which exhibits worse diagnostic performances compared with that of sample entropy imaging. The proposed sample entropy not only outperformed the statistical distribution parameters and Shannon entropy in characterizing hepatic steatosis but also provides the diagnostic ability in clinical fibrosis risk evaluations of individuals with developing hepatic steatosis, as supported by the current findings.

### F. Limitations and Future Work

This study had some limitations. First, the number of patients contributing data for validation of the liver fibrosis detection performance was limited. The inclusion of a larger control group and more patients with different fibrosis scores would increase the reliability of the statistical analysis results. Second, most patients with liver fibrosis also had various grades of hepatic steatosis. Although ultrasound parametric imaging based on sample entropy successfully characterized liver fibrosis in the patients with moderate-to-severe hepatic steatosis, using ultrasound backscattering analysis to detect liver fibrosis in livers without hepatic steatosis or with mild hepatic steatosis is substantially more challenging [32]. Using appropriate surrounding tissues and the corresponding sonographic features as references for standardization of entropy analysis and decomposition of features may be necessary to be further investigated. Third, our recommended computational value set of (WSL, $m$, $r$) for estimating sample entropy is not universally applicable to all systems, transducers, or diseases. Different signal processing procedures (e.g., digital sampling, filtering, or detrending) could affect the time-series pattern and the corresponding sample entropy value [42]. Additional calibrations of the computational settings for sample entropy calculations are necessary for reliable performance in different tissue characterization applications. Finally, RF data that is required for sample entropy imaging but unavailable in most commercial scanners is more objective and beneficial to quantitative analysis because it is independent of demodulation and signal processing in the system. Consequently, functional extension for RF data access in clinical imaging systems should be considered as a critical task in future applications.

## V. Conclusion

Sample entropy is proposed as a new approach to ultrasound parametric imaging for the characterization of hepatic steatosis and fibrosis. We tested the performance of ultrasound parametric imaging with sample entropy by processing raw backscattered RF signals through a sliding-window technique for clinical validation of the results. The results suggest that the set of computational values (WSL = 1 PL, $m =4$, $r =0.1$) maximizes the strength of the correlation between sample entropy and the HFF, as measured through $^{1}\text{H}$-MRS. In particular, sample entropy outperformed conventional Shannon entropy in the assessment of early hepatic steatosis ($\ge $mild); sample entropy could also be used to detect liver fibrosis in individuals with significant hepatic steatosis. The superiority of sample entropy compared with conventional PDF-based entropies may be attributable to the more comprehensive manner in which sample entropy can be used to interpret wave interference in different microstructures through the detection of similarities within the signal waveform and thus be used to assess the irregularity of ultrasound time-series data. Sample entropy imaging proposed in this study is a useful strategy to endow general ultrasound imaging systems with a value-added diagnostic ability in hepatic steatosis assessment and fibrosis risk evaluation.
